# Commentary: Primary Emotional Systems and Personality: An Evolutionary Perspective

**DOI:** 10.3389/fpsyg.2017.01414

**Published:** 2017-08-21

**Authors:** Stefano I. Di Domenico, Richard M. Ryan

**Affiliations:** ^1^Institute for Positive Psychology and Education, Australian Catholic University Sydney, NSW, Australia; ^2^Department of Clinical and Social Sciences in Psychology, University of Rochester Rochester, NY, United States

**Keywords:** big five personality traits, personality neuroscience, basic emotions, SEEKING/PLAY systems, intrinsic motivation

Recent decades have seen growing consensus about the “Big Five” or “Five-Factor” model as a descriptive taxonomy of personality traits (John et al., 2008; McCrae and Costa, 2008). Rather than representing any particular theory, the Big Five traits were identified in factor-analytic investigations of people's descriptions of themselves and others. Having achieved broad agreement for how to describe phenotypic personality traits, researchers have recently intensified efforts on developing explanations for the emergence and functions of the Big Five (e.g., see Fajkowska and DeYoung, 2015).

Personality neuroscience is central to this project, as individual differences in the operation of brain systems, especially emotional systems, have long been a suspected source from which personality traits emerge (e.g., Eysenck, 1967; Gray, 1982; Depue and Collins, 1999; Cloninger, 2000). Montag and Panksepp (M&P) contribute to this endeavor by linking the Big Five to a suite of primary emotional systems (Montag and Panksepp, 2017). Although there are a variety of affective neuroscience models of emotion (e.g., see Sander, 2013), M&P's paper is based on the primary emotional systems described by Panksepp (1998), which are believed to be common in mammals. M&P's cross-species analysis helps situate personality science within a consilient neuroevolutionary framework and, in so doing, affords potential insights into the primordial origins of human personality and how traits function.

For example, M&P link the Big Five dimension of *Agreeableness* to the CARE system, which promotes the affectionate engagement with and nurturing of offspring. Since CARE circuits are stronger in female animals (Panksepp, 1998), the system's alignment with Agreeableness helps make sense of persistent sex differences documented in cross-cultural studies, in which females score about half a standard deviation higher than males on this trait (Lippa, 2010). Similarly, the conspicuous absence of a primary emotional system beneath *Conscientiousness* in M&P's Figure 1 dovetails with studies suggesting that guilt comprises the “affective core” of this trait (Faynard et al., 2012). Faynard et al. (2012) found that Conscientiousness, though negatively associated with the experience of guilt, is positively associated with guilt proneness. While guilt and guilt proneness may have roots in ancient separation-distress systems (Panksepp, 1998, Chapter 14) these emotional dispositions require self-consciousness and therefore depend upon neural capacities beyond primary emotional systems. The fact that M&P's analysis informs empirical findings like the two described above is one reason why their article is compelling.

Nonetheless, M&P's succinct article necessarily leaves many questions unanswered and promising research directions unarticulated. Salient among the questions is the level of resolution researchers might consider when aligning traits with primary emotional systems. The Big Five represent a *hierarchical* taxonomy of traits, with the very broad dimensions of Neuroticism, Extraversion, Openness/Intellect, Conscientiousness, and Agreeableness each subsuming a still indeterminate number of narrower trait dimensions called “facets.” Studies even suggest the existence of intermediate traits called “aspects” (DeYoung et al., 2007). Thus, although we find M&P's Figure 1 to be a useful summary of how primary emotional systems may contribute to trait compositions, some of the systems documented by Panksepp (1998) may be more exactingly aligned with traits at lower levels of the Big Five hierarchy. For example, we might expect PLAY to be primarily associated with *Enthusiasm*, the communal aspect of *Extraversion*, and only secondarily with *Assertiveness*, Extraversion's agentic aspect (DeYoung et al., 2007). Indeed, like PLAY, Enthusiasm has been linked to both dopamine and endogenous opioids, whereas Assertiveness appears to be more strongly associated with dopamine (Allen and DeYoung, 2017). Future studies examining individual differences in primary emotions alongside faceted measures of the Big Five will no doubt foster a more nuanced mapping between the primary emotional systems and the trait hierarchy.

Beyond personality traits, M&P's analysis may also serve as a stepping stone for considering how other universal experiential and behavioral phenomena are based in ancient emotional systems. Our own interest has been in the connection between primary emotional systems and *intrinsic motivation* (Di Domenico and Ryan, 2017). Intrinsic motivation refers to the spontaneous tendency “to seek out novelty and challenges, to extend and exercise one's capacities, to explore, and to learn” (Ryan and Deci, 2000, p.70). When intrinsically motivated, people perform an activity because they find the activity itself interesting or personally satisfying. Though first identified in non-human primates (Harlow, 1950), intrinsic motivation has primarily been studied within the field of human motivation. Intrinsic motivation predicts enhanced performance, learning, and creativity, and it plays an important role in personality development and wellness across the lifespan (Ryan and Deci, 2017). Accordingly, intrinsic motivation is a topic of interest in both basic and applied research.

Intrinsic motivation is used as a broad term to describe activities that are volitionally enacted, growth-promoting, and performed for their own sake. As such, it encompasses activities that are both exploratory (e.g., curiosity, mastery-related behaviors) and socially playful (e.g., sporting activities, social games). Affective neuroscience models of emotion, including Panksepp's (1998), could afford new insights into intrinsic motivation. Distinct systems for exploratory SEEKING and social PLAY suggest different types of intrinsic motivation. Human neuroscience studies have focused on exploratory curiosity and mastery tendencies and we recently made the case that the SEEKING system is a basis for these intrinsically motivated activities (Di Domenico and Ryan, 2017).

Differences between exploratory SEEKING and social PLAY in humans are understudied and thus represent an important direction for intrinsic motivation research. We concur with Panksepp (1998) and others that social PLAY tendencies are a basis for people (especially children) to develop various social competencies and we regard play to be a type of intrinsically motivated socialization. Similar to the ongoing work mapping the primary emotional systems to the Big Five, clarification about the emotional states associated with different types of intrinsically motivated activities is likely to be important. Such studies can also leverage previous experimental work showing that intrinsic motivation is undermined by events that thwart people's feelings of autonomy (volition) and competence (mastery) (Deci et al., 1999). Whether these events similarly undermine exploration, play, and their concomitant affects is an important question for future studies.

## Author contributions

SD conceptualized and wrote the commentary. RR assisted in conceptualizing and writing the commentary.

### Conflict of interest statement

The authors declare that the research was conducted in the absence of any commercial or financial relationships that could be construed as a potential conflict of interest.

## References

[B1] Allen T. DeYoung C. (2017). Personality neuroscience and the five factor model, in The Oxford Handbook of the Five Factor Model, ed Widiger T. A. (Oxford: Oxford University Press).

[B2] Cloninger C. R. (2000). Biology of personality dimensions. Curr. Opin. Psychiatry 13, 611–616. 10.1097/00001504-200011000-00024

[B3] Deci E. L. Koestner R. Ryan R. M. (1999). A meta-analytic review of experiments examining the effects of extrinsic rewards on intrinsic motivation. Psychol. Bull. 125, 627–668. 10.1037/0033-2909.125.6.627 10589297

[B4] Depue R. A. Collins P. F. (1999). Neurobiology of the structure of personality: Dopamine, facilitation of incentive motivation, and extraversion. Behav. Brain Sci. 22, 491–517. 10.1017/S0140525X99002046 11301519

[B5] DeYoung C. G. Quilty L. C. Peterson J. B. (2007). Between facets and domains: 10 aspects of the Big Five. J. Personal. Soc. Psychol. 93, 880–896. 10.1037/0022-3514.93.5.880 17983306

[B6] Di Domenico S. I. Ryan R. M. (2017). The emerging neuroscience of intrinsic motivation. Front. Hum. Neurosci. 11:145. 10.3389/fnhum.2017.00145 28392765 PMC5364176

[B7] Eysenck H. J. (1967). The Biological Basis of Personality. Springfield, IL: Thomas.

[B8] Fajkowska M. DeYoung C. G. (2015). Introduction to the special issue on integrative theories of personality. J. Res. Personal. 56, 1–3. 10.1016/j.jrp.2015.04.001

[B9] Faynard J. V. Roberts B. W. Robins R. W. Watson D. (2012). Uncovering the affective core of conscientiousness: the role of self-conscious emotions. J. Personal. 80, 1–32. 10.1111/j.1467-6494.2011.00720.x PMC3720981 21241309

[B10] Gray J. A. (1982). The Neuropsychology of Anxiety: An Enquiry into the Functions of the Septo-Hippocampal System. New York, NY: Oxford University Press.

[B11] Harlow H. F. (1950). Learning and satiation of response in intrinsically motivated complex puzzle performance by monkeys. J. Comp. Physiol. Psychol. 43, 289–294. 10.1037/h0058114 15436888

[B12] John O. P. Naumann L. P. Soto C. J. (2008). Paradigm shift to the integrative big-five taxonomy: History, measurement, and conceptual issues, in Handbook of Personality: Theory and Research, eds John O. P. Robins R. W. Pervin L. A. (New York, NY: Guilford), 114–158.

[B13] Lippa R. A. (2010). Gender differences in personality and interests: when, where, and why? Soc. Personal. Psychol. Compass 4, 1098–1110. 10.1111/j.1751-9004.2010.00320.x

[B14] McCrae R. R. Costa P. T. Jr. (2008). The five-factor theory of personality, in Handbook of Personality: Theory and Research, eds John O. P. Robins R. W. Pervin L. A. (New York, NY: Guilford), 159–181.

[B15] Montag C. Panksepp J. (2017). Primary emotional systems and personality: An evolutionary perspective. Front. Psychol. 8:464. 10.3389/fpsyg.2017.00464 28443039 PMC5387097

[B16] Panksepp J. (1998). Affective Neuroscience: The Foundations of Human and Animal Emotions. New York, NY: Oxford University Press.

[B17] Ryan R. M. Deci E. L. (2000). Self-determination theory and the facilitation of intrinsic motivation, social development, and well-being. Am. Psychol. 55, 68–78. 10.1037/0003-066x.55.1.68 11392867

[B18] Ryan R. M. Deci E. L. (2017). Self-Determination Theory: Basic Psychological Needs in Motivation Development and Wellness. New York, NY: Guilford Press.

[B19] Sander D. (2013). Models of emotion: The affective neuroscience approach, in The Cambridge Handbook of Human Affective Neuroscience, eds Armony J. Vuilleumier P. (New York, NY: Cambridge University Press), 5–53.

